# The impact of initial antibiotic therapy (linezolid, vancomycin, daptomycin) on hospital length of stay for complicated skin and soft tissue infections

**DOI:** 10.1186/2193-1801-2-696

**Published:** 2013-12-30

**Authors:** Ewa Szczypinska, Alexander Velazquez, Diana Salazar, C Andrew DeRyke, Beata Raczynski, Mark R Wallace

**Affiliations:** Department of Infectious Disease, Orlando Health, Orlando, FL USA; 21 W Columbia St., Suite 102, Orlando, FL 32806 USA

**Keywords:** Vancomycin, Daptomycin, Linezolid, Skin and soft tissue infections

## Abstract

**Background:**

Empiric therapy of inpatient skin and soft tissue infections (SSTIs) generally require methicillin resistant *Staphylococcus aureus* (MRSA) coverage. Limited data are available to directly compare the effect of initial antibiotic choice on treatment outcomes and length of stay (LOS).

**Objective:**

To assess potential differences in length of hospital stay when inpatients with complex skin and soft tissue infections (SSTIs) were initially treated with either vancomycin, linezolid, or daptomycin.

**Methods:**

A retrospective review of 219 patients diagnosed with inpatient SSTI who received linezolid, vancomycin, or daptomycin for >48 hours was performed. Data collected included demographics, comorbidities, microbiologic/laboratory data, additional management (surgical, non-study antibiotics), hospital LOS, treatment outcome and morbidity/mortality.

**Results:**

The three groups evaluated were linezolid (n = 45), vancomycin (n = 90) daptomycin (n = 84). There was no difference between the three groups with respect to gender, age, comorbidities, leukocytosis, fever, antibiotics prior to admission, site of infection culture results and surgical intervention. One death was recorded, not associated with diagnosis of SSTI. No significant difference in LOS was found (P = 0.525) between the 3 groups. The mean LOS in entire cohort was 4.5 days (SD ± 2.5); thirty patients had prolonged LOS for non-SSTI reasons; reanalyzing the data without these 30 patients did not produce any difference in the mean LOS between the 3 groups. Switching vancomycin just prior to discharge to facilitate outpatient therapy was common but did not impact LOS.

**Conclusions:**

No difference was detected in hospital length of stay with respect to the initial choice of antibiotic (linezolid, vancomycin, or daptomycin) for SSTI. The three antibiotic regimens were equally effective in treating SSTIs as judged by LOS, irrespective of age, gender, comorbidities or baseline severity of SSTI. Given the large standard deviation in LOS, this result should be confirmed by larger studies.

## Background

Skin and soft tissue infections (SSTIs) account for a large proportion of hospitalizations. In 2004, approximately 870,000 U.S. hospital admissions were due to SSTIs, an increase of 29% over 2000 data (Edelsbert et al. 2009). Though multiple studies have found varying average inpatient stays and costs for SSTIs, a recent analysis found an average stay of 6.1 days and cost ~6830 United States dollars (USD) per episode (Menzin et al. 2010).

The most common organisms involved in SSTIs are *Staphylococcus aureus* and B-haemolytic streptococci (Rajan 2012). The initial treatment regimen is based on clinical presentation, microbiologic data, hospital antibiogram, physician’s discretion and the pharmacy formulary. Given growing antibiotic resistance, empiric antimicrobial therapies for severe SSTIs must now have MRSA activity, as about half of S. aureus infections are methicillin resistant (Menzin et al. 2010;Rajan 2012). Currently, the leading options for empiric inpatient coverage for SSTIs are linezolid, vancomycin, and daptomycin (Bounthavong et al. 2011). Ceftaroline, telavancin, and tigecycline are newer, but less used, options in this category (van Hal and Paterson 2011).

Daptomycin has superior *in vitro* bactericidal activity against MRSA when compared with vancomycin and linezolid (Maraconescu et al. 2012). Multiple analyses have compared vancomycin (the incumbent “gold standard”) to linezolid and daptomycin. Though “suggestions” of superiority for daptomycin and/or linezolid have been found (van Hal and Paterson 2011;Bounthavong and Hsu 2012;Logman et al. 2010;Davis et al. 2007), the actual value with respect to hospital LOS from changing from initial therapy with vancomycin, a time tested and inexpensive antibiotic, to one of the more expensive newer agents as initial therapy for inpatient SSTIs have not been convincingly demonstrated. As part of a cost benefit analysis at our medical center, we conducted a retrospective study to evaluate the impact of initial inpatient antibiotic choice (daptomycin, linezolid, or vancomycin) on SSTI length of hospital stay.

## Methods

A retrospective cohort review was performed on Orlando Health inpatients that were diagnosed with a skin or soft tissue infection and received one of the three study antibiotics (daptomycin, linezolid, or vancomycin) between January 2009 and September 2010. Charts were selected by ICD-9 codes (680.0 – 686.9) and comprehensively reviewed. One dose of a non-study antibiotic was permitted prior to initiation of therapy with vancomycin, linezolid or daptomycin.

### Inclusion criteria

Patients between the ages of 18–85 with an acute SSTI, defined as three or more of the following: warmth, erythema, swelling, pain, tenderness, lymph node swelling/tenderness, drainage/discharge, or induration, for less than two weeks. Patients were also included if they had an abscess requiring incision and drainage at bedside or in the operating room. Initial antibiotic treatment was daptomycin, linezolid (intravenous or oral), or vancomycin and continued for at least 48 hours. The choice of initial antibiotic was entirely dependent on the attending physician’s preference.

### Exclusion criteria

Patients were excluded if they had osteomyelitis (suspected or proven), decubitus ulcer, necrotizing fasciitis, myositis, gas gangrene, a Gram positive isolate proven resistant to one or more study antibiotics, Gram negative infection, or the presence of concomitant infection upon admission (i.e. pneumonia, UTI).

### Data collection, definitions, statistics

Hospital length of stay was evaluated in relation to the specific antimicrobial regimen chosen on admission. Length of stay was calculated based on nights spent in the hospital. A prolonged stay occurred when patients were kept in hospital beyond the requisite stay for SSTI treatment for unrelated medical or social problems.

Additional data collected included demographics (age, gender), clinical presentation (collected from physician’s progress notes, including antibiotics prior to admission, anatomical site of infection, vital signs, duration of symptoms), co-morbidities (HIV status, diabetes mellitus (DM), peripheral vascular disease (PVD), end stage renal disease (ESRD), immunosuppressive therapy, malignancy), social history (tobacco use), and laboratory/radiological data (white blood cell count, microbiological data, creatinine, imaging). Treatment data collected included surgical/bedside interventions performed, intensive care unit admission, additional non-study antibiotics administered, and whether a switch in therapy occurred. Switching of antibiotic was defined as a switch from study antibiotic to a different study antibiotic or another non-study antibiotic during the hospitalization. Switching of antibiotics was classified into one of the following categories:

1. Transition to outpatient therapy: (changing to oral or simplified once daily intravenous therapy).
2. Treatment failure. No clinical improvement after ≥48 hours; switched to a different antibiotic based on physician’s judgment.
3. De-escalation to a B-lactam antibiotic after organism identification and susceptibility available (i.e. methicillin susceptible *Staphylococcusaureus* or Group A streptococci).
4. Allergy or adverse reaction.
5. Therapeutic preference of the attending physician.

Descriptive statistics for all data were collected and stored using Microsoft Excel software. Statistical tests were performed using SPSS software with a p value < 0.05 used to define statistical significance. Nominal data were compared by using the Pearson *X*^2^ test. Age and length of stay measures were analyzed using an ANOVA.

This research was approved through the Institutional Review Board (IRB) at Orlando Health.

## Results

**Figure 1:**
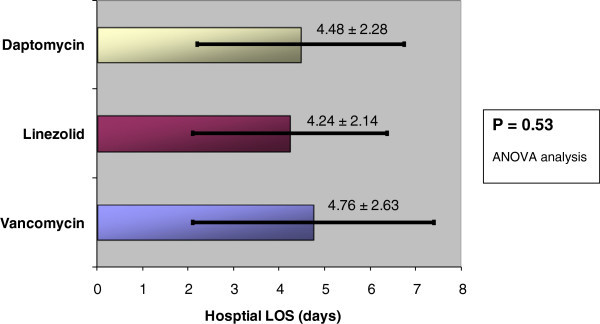
**Hospital length of stay (LOS) (N = 219).**

**Figure 2:**
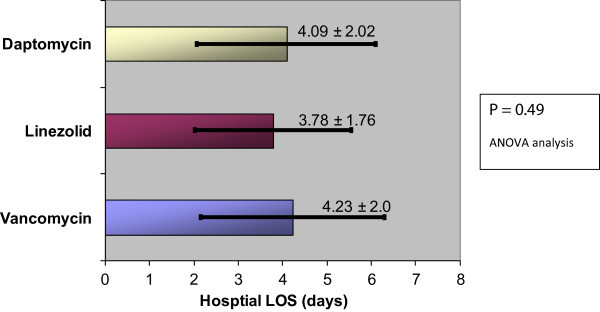
**Patients with prolonged LOS for non-SSTI reasons eliminated (N = 189).**

Four hundred and seventy two charts were reviewed; a total of 219 patients met the inclusion criteria (daptomycin n = 84, linezolid n = 45, and vancomycin n = 90). No difference in hospital length of stay was observed regardless of initial antibiotic choice for patients admitted to the hospital with skin and soft tissue infection (p = 0.53) (Figure 1). The mean LOS in entire cohort was 4.5 days (SD ± 2.5). Thirty patients had a prolonged hospital length of stay beyond what was required for treatment of their SSTI; the data with these patients excluded also showed no difference in LOS: (n = 189, p = 0.49) (Figure 2).

The mean age in the daptomycin, linezolid and vancomycin groups was 49, 48, and 52 years (SD+/- 2.5), respectively. There was no difference in gender (p = 0.64), history of intravenous drug (p = 0.90) or tobacco use (p = 0.26). The clinical presentation was not significantly different between the three antibiotic groups when evaluated by preadmission antibiotics, site of infection culture results, adenopathy, fever, leukocytosis, drainable foci, radiological evidence of infection, or requirement for surgical intervention (Table 1). Furthermore, except for end stage renal failure which was over represented in the daptomycin group (p = 0.038), there was no difference between observed co-morbidities including diabetes, heart failure, immunosuppressive drugs, HIV, peripheral vascular disease, history of malignancy, history of transplant, or previous known colonization with MRSA (Table 2). Vancomycin levels were collected in 44 cases (49%), with trough levels >/= 10 mg/L in 87% cases (n = 38).

**Table 1:** **Clinical and microbiologic features of SSTIs**

	Daptomycin	Linezolid	Vancomycin	P value
Antibiotics prior to admission	29 (34)	11 (24)	20 (22)	0.16
Adenopathy	6 (7)	0 (0)	9 (10)	0.09
Fever	14 (16)	7 (15)	20 (22)	0.53
Leukocytosis	53 (63)	20 (44)	46 (51)	0.09
Drainable Foci	34 (40)	16 (35)	38 (42)	0.75
Coadministration non-study antibiotic	64 (76)	32 (71)	76 (84)	0.16
Radiological evidence of collection/abscess	10 (11)	2 (4)	12 (13)	0.27
Wound + MRSA	26 (31)	13 (29)	28 (31)	
Wound + MSSA	7 (8)	7 (16)	7 (8)	
Positive blood cultures*	2 (2)	0	4 (4)	

*All positive blood cultures were MRSA.

n (%).

Pearson *X* ^*2*^ test analysis.

**Table 2:** **Comorbidities of SSTI patients**

	Daptomycin	Linezolid	Vancomycin	P value
Diabetes	24(28)	9(20)	23(25)	0.56
Heart failure	1(1)	3(6)	3(3)	0.24
ESRD	7(8)	0(0)	2(2)	0.03
Immunosuppressive therapy	4(4)	1(2)	5(5)	0.67
HIV	1(1)	3(6)	6(6)	0.16
Peripheral vascular disease	6(7)	3(6)	4(4)	0.73
History malignancy	5(6)	1(2)	11(12)	0.09
History transplant	1(1)	1(2)	3(3)	0.63
Colonized MRSA	9(10)	5(11)	9(10)	0.97

n (%).

Pearson *X* ^*2*^ test analysis.

There was a significant difference in switching of antibiotics during treatment. The vancomycin group had the highest percentage of switching (55%), followed by daptomycin (19%), and linezolid (11%) (p < 0.05). The most common reason for switching antibiotics in all groups was a transition to outpatient therapy; 88% of vancomycin changes were to either daptomycin, so as to facilitate once daily outpatient intravenous antibiotic therapy, or a transition to oral treatment. The vancomycin patients changed to daptomycin almost invariably occurred on the last hospital day and had no influence on LOS. Only one patient in any group (daptomycin) was changed due to treatment failure (Table 3). No patient was excluded or switched because of Gram positive resistance.

**Table 3:** **Reason antibiotics switched during treatment**

	Daptomycin (16)	Linezolid (5)	Vancomycin (50)
De-escalation	3	1	4
Transition to outpatient	11	2	44
Presumed treatment failure	1	0	0
Allergy or adverse effect	0	1	1
Therapeutic preference	1	1	1

One death was recorded, not associated with the diagnosis of SSTI.

## Discussion

This retrospective “real-world” study found no significant differences in length of stay between the three most commonly used empiric inpatient anti-MRSA antibiotics for skin and soft tissue infections requiring inpatient care. Previous in vitro work supports daptomycin as the superior agent (Maraconescu et al. 2012). Multiple clinical studies, analyses and reviews, have suggested a possible advantage for linezolid (Menzin et al. 2010;Bounthavong et al. 2011;van Hal and Paterson 2011;Bounthavong and Hsu 2012;Logman et al. 2010;Itani et al. 2012;Barron et al. 2012;Watkins et al. 2012) or daptomycin (Itani et al. 2010;Quist et al. 2012;Falcone et al. 2012;Bliziotis et al. 2010;Davis et al. 2007) as compared to vancomycin in terms of time to clinical cure, hospital length of stay, or both. Despite the evidence favoring linezolid or daptomycin over vancomycin, some authors have been unconvinced of the clinical relevance of the purported superiority of the newer agents (Eckmann and Dryden 2010). We chose to focus on LOS as a single key parameter of efficacy as it is closely tied to hospital cost and is more straightforward than assessment of clinical parameters such as time of resolution of cellulitis, etc.

Vancomycin’s main advantages are its 50 year track record and low cost, but its use requires careful monitoring to avoid nephrotoxicity (van Hal and Paterson 2011). Though overt resistance remains rare, some institutions have noted “MIC Creep,” a situation in which vancomycin MICs ≥1.5 mg/l are found with increasing frequency. Such isolates may be difficult to cure with vancomycin, even at higher doses (Brink 2012). At our institution, such isolates comprise <1.5% of all *Staphylococcus aureus* isolates using the Vitek 2 methodology (BioMerieux). Daptomycin is a remarkably easy drug to use, but it may occasionally cause myopathy or serious eosinophilic pneumonia (van Hal and Paterson 2011). A larger concern is the increasing frequency of daptomycin resistance (van Hal and Paterson 2011;van Hal et al. 2011;Velazquez et al. 2013). Linezolid is the simplest of the three antibiotics, given its high oral bioavailability and simple intravenous to oral conversion, but its use may be complicated by thrombocytopenia, peripheral neuropathy and drug interactions with selective serotonin inhibitors (Rajan 2012). Resistance to linezolid remains rare, but may be increasing (van Hal and Paterson 2011).

This retrospective study has limitations. The patients were allocated into groups by the preferences of the attending physicians, many of whom held strong views on the relative superiority of one agent vs. the others. Attendings may have started “sicker” patients on the non-vancomycin regimens, though we have no evidence this occurred as the groups were comparable with respect to demographics, admission WBC, fever and baseline co-morbidities. Institutions with diminishing vancomycin susceptibility may experience poorer outcomes with vancomycin use. The study may have been underpowered to detect differences; larger numbers might have turned the trend in favor of linezolid into a statistically significant finding.

Vancomycin remains a key antibiotic in the gram positive armamentarium. We found no evidence for any significant difference in length of stay for SSTI between the three common initially used inpatient agents, and are continuing to advise clinicians at our medical center to start with vancomycin for complicated inpatients SSTIs, (unless there were contraindications), both to limit cost and the risk of daptomycin or linezolid resistance. How long vancomycin will remain the workhorse inpatient choice remains unclear, and will likely depend on differential resistance among these 3 agents, new drugs such as ceftaroline, and external economic factors.
